# Tea Tree Essential Oil Enriches Lactic Acid Bacteria and Improves Paper Mulberry Silage Quality

**DOI:** 10.3390/microorganisms14091957

**Published:** 2026-09-04

**Authors:** Xinyi Chen, Sijin Guo, Xiaokai Zheng, Yingchao Sun, Rongzheng Huang, Yongcheng Chen, Chunhui Ma, Fanfan Zhang

**Affiliations:** 1 College of Animal Science & Technology, Shihezi University, Shihezi 832000, China; cxyshzu@outlook.com (X.C.); gsjshzu@outlook.com (S.G.); zhengxiaokaiyue@163.com (X.Z.); 17633866730@163.com (Y.S.); chenyonchn@163.com (Y.C.); chunhuima@126.com (C.M.); 2 Bingtuan Key Laboratory for Efficient Utilization of Non-Grain Feed Resources, Shihezi 832000, China

**Keywords:** essential oil, *Broussonetia papyrifera*, silage, fermentation quality, bacterial community

## Abstract

Paper mulberry (*Broussonetia papyrifera*) is a high-protein forage, but its high buffering capacity and low water-soluble carbohydrate (WSC) content hinder silage fermentation. This study evaluated the effect of tea tree (Melaleuca alternifolia) essential oil (TTO) as a natural additive for paper mulberry silage. TTO was added at 0 (CK), 500 (CSD), and 1000 mg/kg (CSG) fresh weight, with samples collected on days 7, 15, 30, and 90. After 90 days, CSD had the lowest pH and the highest lactic acid content. NH_3_-N/TN was significantly lower than in CK (*p* < 0.05), and the retention of dry matter, crude protein and water-soluble carbohydrate was improved (*p* < 0.05). Compared with CK, CSD exhibited the highest lactic acid bacteria (LAB) counts, along with the lowest mold and aerobic bacterial counts (*p* < 0.05). However, CSG did not further improve fermentation quality, and most of the indicators were not significantly different from CSD (*p* > 0.05). The in vitro gas production test showed that CSD significantly increased the theoretical maximum gas production and the maximum gas production rate (*p* < 0.05). Additionally, 16S rRNA sequencing revealed a distinct bacterial succession in CSD, which enriched beneficial *lactic acid bacteria* (LAB) such as *Pediococcus* while outcompeting less competitive taxa. This microbial reshaping redirected the community toward acidification and nutrient retention. Collectively, these results demonstrate that TTO at 500 mg/kg is a promising natural additive for improving paper mulberry silage quality through selective reshaping of the bacterial community.

## 1. Introduction

The projected 50% increase in global food demand by 2050 presents a formidable challenge for sustainable agricultural systems [1,2], intensifying pressure on feed resources and widening the supply–demand gap for conventional forages [3]. In many regions, forage systems are dominated by limited species such as alfalfa (*Medicago sativa*) and oat (*Avena sativa*), leading to reduced agrobiodiversity and constrained nutritional provision for livestock production [4,5].

Paper mulberry (*Broussonetia papyrifera*), a perennial deciduous tree of the Moraceae family, is native to East Asia and has been widely cultivated and occasionally naturalized across Southeast Asia, the Pacific Islands, and parts of Africa and the Americas [6]. Its remarkable adaptability to diverse climatic and edaphic conditions, coupled with rapid growth and high biomass yield, has drawn attention to its potential as a forage resource. However, these same traits have also led to its documentation as an invasive or potentially invasive species in some introduced regions, where unmanaged spread in disturbed habitats can occur [7]. It is important to recognize that in its native range and under cultivated systems with regular harvesting regimes, such as the silage production system employed in the present study, its spread is effectively controlled. In fact, the very characteristics that underlie its reported invasiveness, namely vigorous regrowth after cutting, tolerance to drought and poor soils, and high productivity, are precisely the agronomic attributes that make it a valuable candidate for sustainable forage production on marginal lands. Accordingly, paper mulberry has emerged as a promising alternative forage due to its high crude protein content (>20%), rich amino acid profile, and substantial biomass yield [8,9]. Its strong adaptability to drought and marginal soils underscores its dual value for ecological restoration and forage production [10]. Studies have shown that paper mulberry silage improves growth performance, milk composition, and antioxidant capacity in ruminants [8,11], and enhances laying performance and intestinal health in monogastric animals [12,13]. Furthermore, it has been reported to improve rumen fermentation and modulate microbial communities in dairy heifers [14].

Ensiling paper mulberry, however, presents significant challenges. Its high crude protein, low water-soluble carbohydrates (WSC), and high buffering capacity impede rapid pH decline during fermentation, which favors the proliferation of undesirable microorganisms such as clostridia and molds [15] and leads to increased nutrient losses and elevated ammonia nitrogen concentrations [16]. Although wilting, mixing with energy-rich feeds, and microbial inoculants have been employed to address these issues [15,17], their efficacy remains variable.

Essential oils (EOs) have attracted attention as natural antimicrobial agents in animal nutrition [18]. Their antibacterial activity arises from multi-target mechanisms, including disruption of microbial membrane integrity, enzyme inhibition, and interference with quorum sensing [19,20,21]. Notably, EOs exhibit selective antimicrobial properties: minimum inhibitory concentrations (MICs) against lactic acid bacteria (LAB) are substantially higher than against spoilage organisms such as molds and clostridia [22,23]. This characteristic makes them attractive as silage additives that can suppress undesirable microbes without compromising LAB-mediated fermentation [24].

Evidence indicates that various EOs improve silage fermentation quality and aerobic stability. Cumin and lemongrass oils enhanced fermentation quality by promoting LAB proliferation [25,26,27], whereas oregano-, cinnamon-, and carvacrol-containing compounds effectively inhibited yeast growth and reduced protein degradation [28,29]. More recently, *Amomum villosum*, oregano residues, and pine needle oil have been reported to modulate bacterial communities and suppress spoilage in silage [30,31,32,33]. During successful ensiling [34], the microbial community shifts from a taxonomically diverse epiphytic microbiota to a Lactobacillus-dominated community characterized by reduced evenness and species richness, which is reflected by decreased Shannon and Chao1 indices [35]. Monitoring these metrics is critical for evaluating whether EO treatment selectively enriches beneficial LAB or promotes a more homogeneous community structure.

Among the various EOs, tea tree essential oil (TTO) shows considerable potential for silage application. TTO is primarily composed of terpinen-4-ol (30–40%), gamma-terpinene (10–28%), and alpha-terpinene (5–13%), which confer broad-spectrum antimicrobial activity [18]. This activity is mainly attributed to terpinen-4-ol, which disrupts microbial membrane integrity through its lipophilic nature [20,36]. Its multi-component composition also reduces the risk of resistance development compared to conventional antibiotics [20]. Previous studies have reported that TTO exhibits MIC values of 1% to 2% (*v*/*v*) against *Lactobacillusstrains* [20], with an MBC of 2% (*v*/*v*) [37]. This concentration difference means that at the levels effective for inhibiting spoilage microbes, the activity of LAB is not significantly affected. Research on sorghum silage found that TTO at 1000 mg/kg, combined with rosemary and citronella oils, increased LAB counts [37]. However, the standalone efficacy of TTO in high-protein, low-WSC forages such as paper mulberry has not been systematically evaluated.

A preliminary screening experiment was conducted to compare seven EOs at two concentrations for their effects on paper mulberry silage quality. TTO exhibited the most favorable fermentation profile among these Eos. The present study further investigated the effects of TTO on the dynamics of fermentation quality and microbial community composition of paper mulberry silage, to lay a theoretical foundation for the subsequent development of essential oil-based silage additives.

## 2. Materials and Methods

### 2.1. Plant Material and Experimental Site

Paper mulberry (*Broussonetia papyrifera*) was cultivated at the 16th Experimental Base of the Agricultural Science Research Institute, Seventh Agricultural Division, Xinjiang Production and Construction Corps (44°20′ N, 83°51′ E, 450 m above sea level), China. The site has a temperate continental climate with an annual mean temperature of 7.6 °C. The soil salt content was 0.38%. Paper mulberry was sown in April 2022 using film-mulched hole sowing at approximately 24,000 plants/ha. Whole-plant material was harvested on 15 September 2023, at approximately 1.5 m height with a stubble height of 5–10 cm, chopped to 1–2 cm, and stored for subsequent ensiling. The nutritional composition of the raw material is presented in Table 1.

### 2.2. Preliminary Screening of Essential Oils

A preliminary experiment was conducted to identify the optimal essential oil for paper mulberry silage. Seven essential oils (pine needle, Sichuan pepper, Thymus vulgaris, tea tree, Origanum vulgare, rose, and Cuminum cyminum) were each tested at 500 and 1000 mg/kg (fresh weight basis), with distilled water as control, comprising 15 treatments. Paper mulberry was ensiled for 90 days, after which nutritional quality (dry matter, DM; crude protein, CP; water-soluble carbohydrates, WSC; neutral detergent fiber, NDF; and acid detergent fiber, ADF), fermentation characteristics (pH, lactic acid, LA; acetic acid, AA; propionic acid, PA; and ammonia nitrogen, NH_3_-N), and microbial populations (lactic acid bacteria, LAB; molds; yeasts; and aerobic bacteria, AB) were assessed. TTO at 500 mg/kg exhibited the most favorable fermentation profile (lowest terminal pH, highest lactic acid, and lowest mold count) among all treatments. Detailed screening data are provided in Supplementary Table S1. Based on these results, TTO was selected for the subsequent in-depth study.

### 2.3. Essential Oils

The tea tree essential oil (*Melaleuca alternifolia*, CAS No. 68647-73-4) used in this study was purchased from Shanghai Yuanye Biotechnology Co., Ltd. (Shanghai, China), at the current international standard for terpinen-4-ol type TTO. According to the European Chemicals Agency (ECHA) and the Scientific Committee on Consumer Safety (SCCS), TTO with this CAS number is recognized as conforming to the specifications of ISO 4730:2017 for the terpinen-4-ol type, with terpinen-4-ol (35–48%), γ-terpinene (14–28%), α-terpinene (6–12%), and 1,8-cineole (traces-10%) as its major constituents. The essential oil was stored at 4 °C in amber glass bottles until use.

### 2.4. Experimental Design and Silage Preparation

A single-factor completely randomized design was used with three treatments, CK (control, no additive), CSD (TTO at 500 mg/kg), and CSG (TTO at 1000 mg/kg), where ‘D’ and ‘G’ denote the low (500 mg/kg) and high (1000 mg/kg) addition levels.

Chopped paper mulberry was mixed with either distilled water (control) or the respective concentration of TTO (CSD and CSG). Approximately 500 g of each mixture was packed into a polyethylene silage bag (35 cm × 45 cm, 0.2 mm) with a one-way air valve. Bags were compacted, vacuum-sealed, and stored at room temperature in the dark. Each treatment had 3 replicates, with 5 bags per replicate. Samples were collected at 0, 7, 15, 30, and 90 days of ensiling.

### 2.5. Sample Collection and Quality Analysis

Silage bags were opened at 7, 15, 30, and 90 days. A total of 20 g of each sample was collected by the quartering method, mixed with 180 mL distilled water in a 250 mL conical flask, and extracted at 4 °C for 24 h. The extract was filtered through four layers of gauze and filter paper, then stored at −20 °C for fermentation quality analysis.

Another 100 g of each sample was dried at 105 °C for 30 min and then at 65 °C to constant weight. This material was used for dry matter and nutritional quality analysis.

Referring to the AOAC (2023) methods [38], DMcontent was determined by oven drying at 105 °C to constant weight (method 930.15) and CP was measured by the Kjeldahl method using copper sulfate as a catalyst (method 984.13). NDF and ADF were analyzed following the methods of Van Soest et al. [39], with the NDF assay including heat-stable *α*-amylase. WSCs were quantified using the anthrone colorimetric method [40].

Extract pH was measured with a pH meter. Ammonia nitrogen was determined by the phenol-sodium hypochlorite colorimetric method. Lactic acid, acetic acid and propionic acid were analyzed by HPLC (Agilent 1260) [41].

For microbial enumeration, 10 g of silage was homogenized in 90 mL of sterile saline (0.85% NaCl) and serially diluted (10^−1^ to 10^−7^). An aliquot of 100 µL from each appropriate dilution was evenly spread onto the surface of selective agar plates using the spread plate method, with three replicate plates prepared for each dilution. LAB were enumerated on de Man, Rogosa and Sharpe agar (PM0560) after anaerobic incubation at 36 °C for 48 h, and aerobic bacteria were enumerated on nutrient agar (PM0670) after aerobic incubation at 36 °C for 48 h. Molds and yeasts were enumerated on Rose Bengal agar medium (PM0631) after incubation at 28 °C for 120 h. Colonies (30–300 per plate) were counted, and results were expressed as log_10_ CFU/g fresh weight. All media were purchased from Coolaber (Beijing, China). Enumeration followed GB 4789.35-2016 [42], GB/T 4789.2-2003 [43] and GB 4789.15-2016 [44].

### 2.6. In Vitro Gas Production

All experimental procedures involving animals were approved by the Biological Ethics Committee of Shihezi University (approval number: A2025-178; March 2025). Efforts were made to minimize animal distress during rumen fluid collection, which was performed by trained personnel. The health and behavior of the animals were monitored daily throughout the experiment.

Three Altay sheep (*Ovis aries*) with similar body weight were selected as rumen fluid donors. The animals were housed in individual pens under standard management conditions at Shihezi University (Shihezi, China). The donor sheep had been maintained on a long-term basis on a total mixed ration (TMR) formulated to meet or exceed the nutrient requirements for growing sheep according to the National Research Council (NRC) guidelines. Fresh water was available ad libitum throughout the experimental periods. Rumen fluid was collected from each animal 1 h before morning feeding, pooled, filtered through four layers of cheesecloth, and transferred to a pre-warmed CO_2_-flushed container. Mixed medium was prepared using the Menke method [45] by combining artificial rumen fluid and rumen fluid at a 2:1 (*v*/*v*) ratio under continuous CO_2_. Air-dried samples (0.2 g) were weighed into glass syringes. Each syringe received 30 mL of the mixed medium, was sealed with petroleum jelly, and incubated immediately at 39 °C. Each treatment and blank were run in triplicate. Gas production was recorded at 0, 2, 4, 6, 8, 10, 12, 24, 48, and 72 h for gas kinetic parameter calculation.

### 2.7. Bacterial Community Analysis

Based on the screening results, CSD (500 mg/kg TTO) outperformed CSG across multiple indicators; therefore, CSD and the control (CK) at 90 days were selected for 16S rRNA sequencing. Frozen samples stored at −80 °C were transported to Majorbio Bio-Pharm Technology Co., Ltd. (Shanghai, China), on dry ice for analysis. Total genomic DNA was extracted from 0.3 g of each frozen sample. The V3-V4 hypervariable region of the bacterial 16S rRNA gene was amplified using the universal primers 338F (5′-ACTCCTACGGGAGGCAGCAG-3′) and 806R (5′-GGACTACHVGGGTWTCTAAT-3′) [46]. The purified amplicons were pooled at equimolar concentrations and subjected to paired-end sequencing (2 × 300 bp) on the Illumina platform. DNA extraction, PCR amplification, library construction, and sequencing were completed by Majorbio Bio-Pharm Technology Co., Ltd. (Shanghai, China).

### 2.8. Statistical Analysis

All data were analyzed using SPSS 26.0 (IBM Corp., Armonk, NY, USA). A two-way analysis of variance (ANOVA) followed by Duncan’s multiple range test was first performed to assess the main effects of treatment, fermentation time, and their interaction. Based on the screening results, the optimal treatment (CSD, 500 mg/kg TTO) and the control (CK) were further compared at each storage time by one-way ANOVA followed by Duncan’s multiple range test. Statistical significance was set at *p* < 0.05. The gas production parameters were calculated using a one-variable nonlinear regression model (Gompertz model) in DPS V7.05 software, following the equation:

(1) *X*_2_ = *C*_1_ × exp [−*C*_2_ × exp (−*C*_3_ × *X*_1_)]

(2) *λ* = [ln(*C*_2_) − 1]/*C*_3_

(3) *Tmax* = ln(*C*_2_)/*C*_3_

*X*_2_ is the gas production (mL); *C*_1_ is the theoretical maximum gas production (mL·200 mg^−1^ DM); *C*_2_ is the gas production rate parameter; *C*_3_ is the gas production rate constant (h^−1^); *λ* is the lag time (h); and *Tmax* is the time to reach the maximum gas production rate (h).

Raw paired-end reads were quality-filtered using fastp (v0.23.4) [46]: bases with a quality score below 20 at the 3′ end were trimmed with a 50 bp sliding window; reads shorter than 50 bp after trimming or containing more than five N bases were discarded. Quality-filtered reads were merged into contigs using FLASH (v1.2.11) [47], with a minimum overlap length of 10 bp and a maximum mismatch ratio of 0.2 in the overlapping region. Samples were demultiplexed by barcode (zero mismatches allowed) and primer sequences (maximum two mismatches allowed), and reads were oriented accordingly. Merged sequences were clustered into operational taxonomic units (OTUs) at 97% sequence identity using USEARCH (v11) [48,49], and chimeric sequences were removed. Taxonomic classification was performed using the RDP Classifier (v2.11) against the Silva 138.2/16S_bacteria database at a confidence threshold of 70% [50]. Sequences annotated as chloroplast or mitochondrial were removed. All samples were rarefied to 33,486 reads per sample.

Alpha diversity indices (Shannon and Chao1) and beta diversity (PCoA based on Bray–Curtis distance) were calculated [51]. Differential taxa were identified by LEfSe (LDA > 2.0). Spearman correlation and redundancy analysis (RDA) were used to assess relationships between bacterial genera and fermentation parameters.

## 3. Results

### 3.1. Nutritional Quality of Paper Mulberry Silage

The nutritional composition of paper mulberry silage was significantly affected by both treatment and ensiling duration (Table 2), with a significant treatment-by-time interaction observed only for DM and NDF (*p* < 0.05). During ensiling, DM, CP, and WSCs generally declined, with CSD generally maintaining the highest values. NDF and ADF also decreased over time, with treatment differences emerging at different time points, from day 30 for NDF and from day 15 for ADF. At 90 days, CSD exhibited significantly higher DM (25.55%), CP (19.30%), and WSCs (5.13%) than CK (21.79%, 17.32%, and 3.65%; *p* < 0.05).

### 3.2. Fermentation Characteristics

Fermentation characteristics are summarized in Table 3. TTO (TTO) significantly improved the fermentation quality of paper mulberry silage. Across all treatments, pH declined with ensiling time (*p* < 0.001), while lactic acid accumulated progressively (*p* < 0.001). From day 15 onwards, CSD exhibited lower pH and higher LA than CK and CSG (*p* < 0.05), reaching 4.20 for pH and 5.63% DM for LA at day 90, compared with 5.64 and 3.72% for CK, respectively.

Ammonia nitrogen increased during ensiling (*p* < 0.001). CSD and CSG showed lower NH_3_-N/TN ratios than CK from day 15 onward (*p* < 0.05), with CSD at 9.20% and CK at 14.42% at day 90. Acetic and propionic acids also increased over time (*p* < 0.001), with CSD and CSG exhibiting higher concentrations than CK, reaching significance for CSD at day 30 and day 90, and for CSG at day 90 (*p* < 0.05).

### 3.3. Microbial Populations

Microbial populations are summarized in Table 4. All microbial groups showed significant treatment × time interactions (*p* < 0.001). LAB counts declined in CK but increased in CSD and CSG over the 90-day period. CSD maintained higher LAB counts than CK from day 15 onward (*p* < 0.05) and exceeded CSG at day 90. Mold counts decreased across all treatments, with CSD remaining the lowest throughout. Yeast counts increased over time in all groups and converged by day 90, with no significant differences among treatments. AB declined with ensiling time; both CSD and CSG were below CK from day 30 onward (*p* < 0.05).

### 3.4. In Vitro Gas Production Kinetics

**Figure 1:**
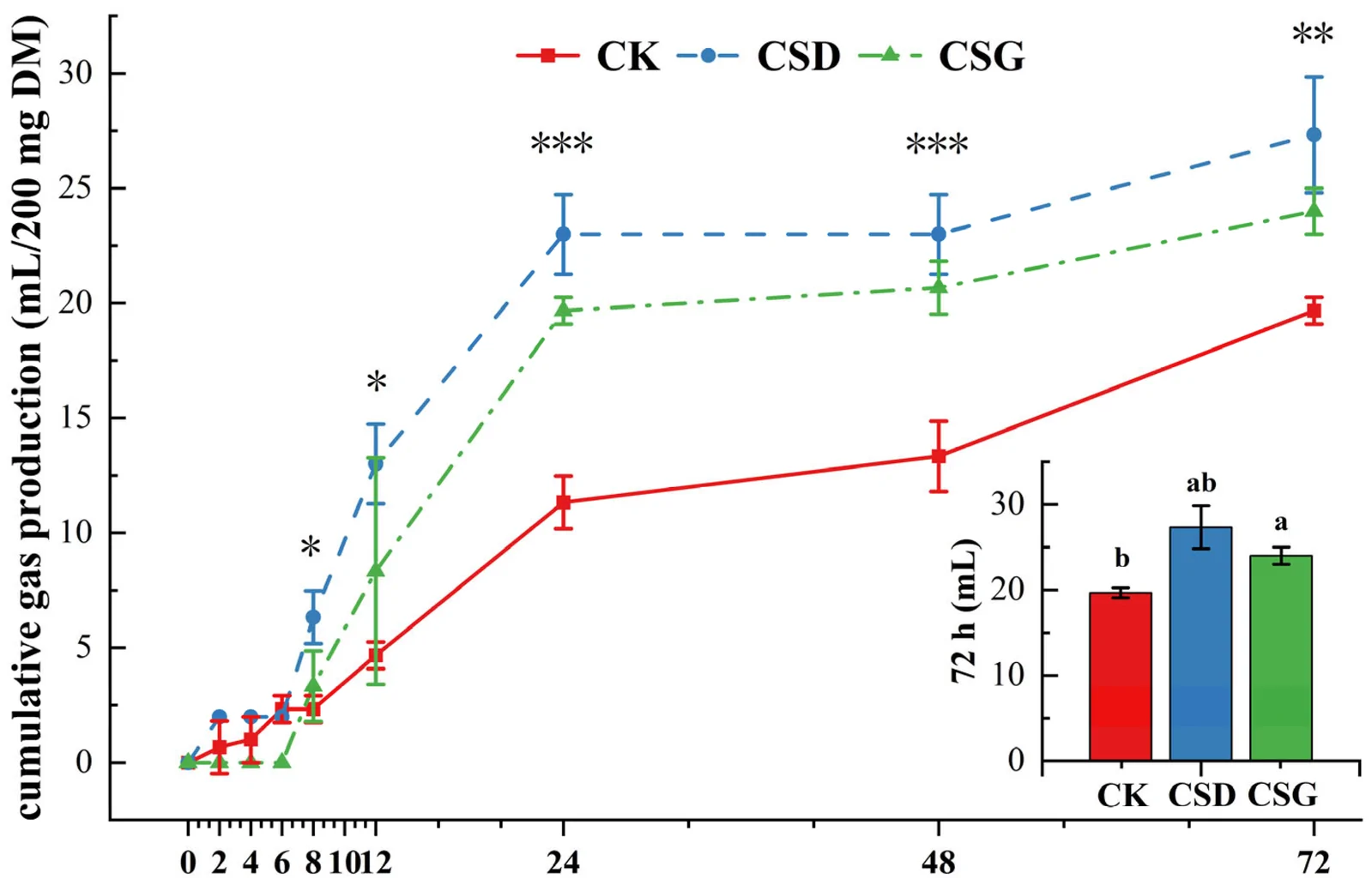
Gompertz model fitting of in vitro gas production kinetics of paper mulberry silage treated with different concentrations of TTO. Cumulative gas production over 72 h is shown for the CK (control), CSD (500 mg/kg TTO), and CSG (1000 mg/kg TTO) groups. The inset bar chart displays the final gas production at 72 h. Values are presented as means ± SD. Asterisks indicate significant differences among treatments at each time point (*** *p* < 0.001, ** *p* < 0.01, * *p* < 0.05). Different lowercase letters above bars denote significant differences in 72 h gas production (*p* < 0.05).

Cumulative gas production over the 72 h incubation period is shown in Figure 1. During the first 6 h, gas accumulation remained minimal across all treatments, with CSD showing a slight early response (2.00 mL at 2 h), whereas CK produced only 0.67 mL at 2 h and CSG remained at zero until 6 h.

From 8 h onward, treatment differences emerged, with CSD consistently producing the most gas, followed by CSG and then CK. This ordering persisted through 72 h, at which point cumulative gas production reached 27.33 mL for CSD, 24.00 mL for CSG, and 19.67 mL for CK. The inset in Figure 1 provides a direct comparison of 72 h values.

The coefficient of determination (*R*^2^) for the Gompertz model fitting of each treatment group exceeded 0.92 across all replicates, indicating a good fit to the cumulative gas production data. Gompertz model parameters are presented in Table 5. Significant differences in gas production kinetic parameters were observed among treatment groups. The maximum theoretical gas production (*C*_1_) was highest in the CSD treatment, significantly greater than in CK and CSG (*p* < 0.001). The *C*_2_ parameter was highest in the CSG treatment, markedly exceeding that in CSD and CK (*p* = 0.020). For the maximum gas production rate (*C*_3_), both CSD and CSG treatments exhibited significantly higher values than CK (*p* = 0.024). The lag time (*λ*) was longest in the CSG treatment, substantially longer than in CK and CSd (*p* = 0.005). A similar trend was observed for *Tmax*, with the CSG treatment peaking significantly later than CK and CSD (*p* = 0.007).

### 3.5. Bacterial Community Diversity and Composition

Based on the comprehensive evaluation of fermentation quality (pH, NH_3_-N/TN, LA, AA, PA), nutrient preservation (DM, CP, WSC), and microbial populations (LAB, mold, yeast, AB), the CSD treatment outperformed CSG. Consequently, only CK and CSD were selected for subsequent 16S rRNA gene sequencing to investigate the bacterial community shifts associated with TTO supplementation.

After quality filtering, a total of 1,787,520 high-quality sequences were obtained from 40 samples, with an average length of 424 bp per sequence. The number of sequences per sample ranged from 40,358 to 64,489. All samples were rarefied to 33,486 sequences for downstream analysis, yielding 493 OTUs.

#### 3.5.1. Alpha and Beta Diversity

**Figure 2:**
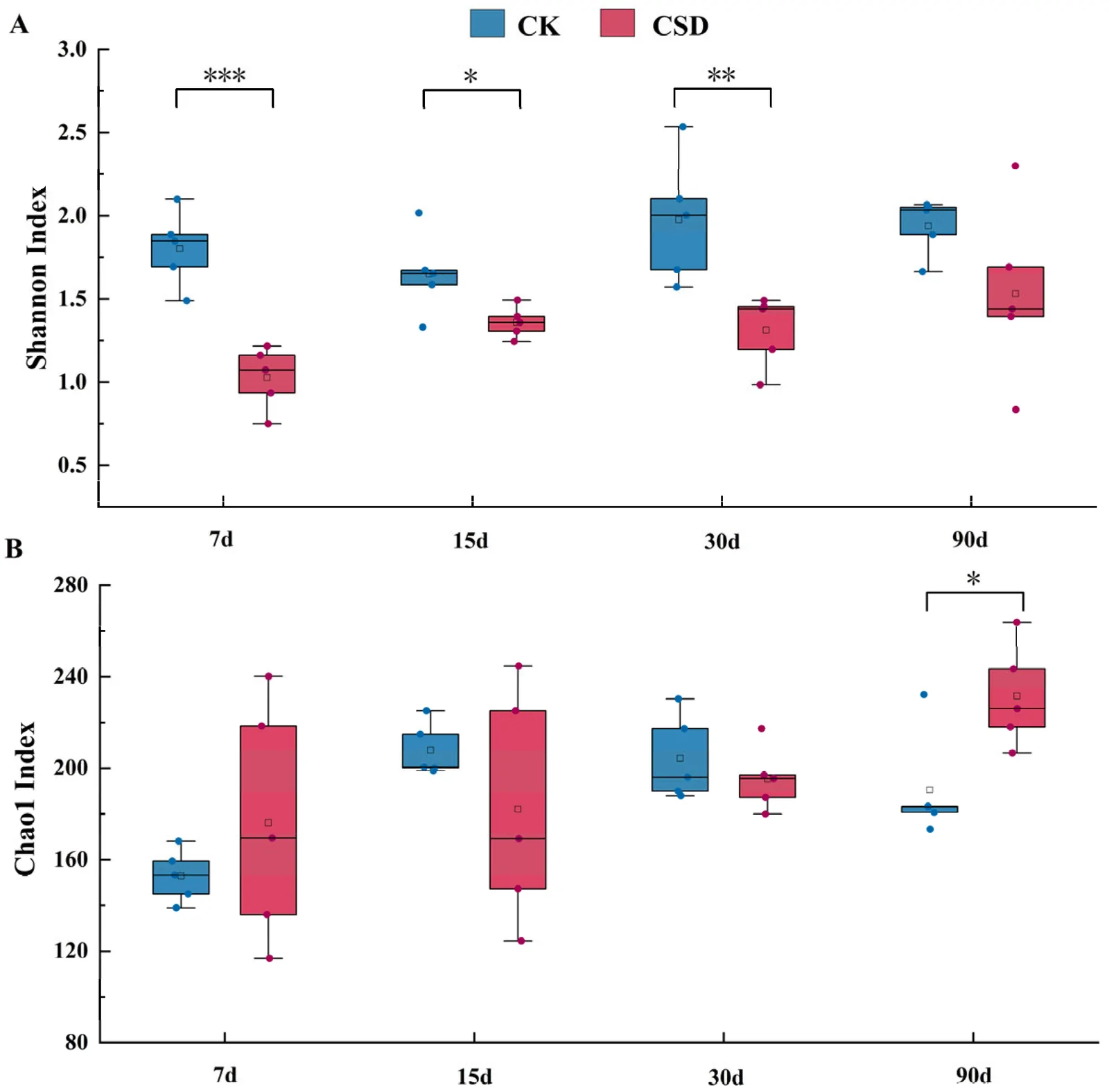
Alpha diversity indices of bacterial communities in paper mulberry silage treated with or without TTO. (**A**) Shannon; (**B**) Chao1. CK was control group and CSD was 500 mg/kg TTO; 7 d, 15 d, 30 d, and 90 d were ensiling time (7, 15, 30, and 90 d). Statistical significance was assessed by Duncan’s multiple range test. * *p* < 0.05, ** *p* < 0.01, *** *p* < 0.001.

Alpha diversity indices are presented in Figure 2. Shannon diversity was higher in CK than CSD at 7, 15, and 30 d (*p* < 0.05), but not at 90 d (*p* = 0.140). Chao1 richness did not differ between treatments through 30 d; by 90 d it was higher in CSD (*p* = 0.023).

#### 3.5.2. Community Overlap

**Figure 3:**
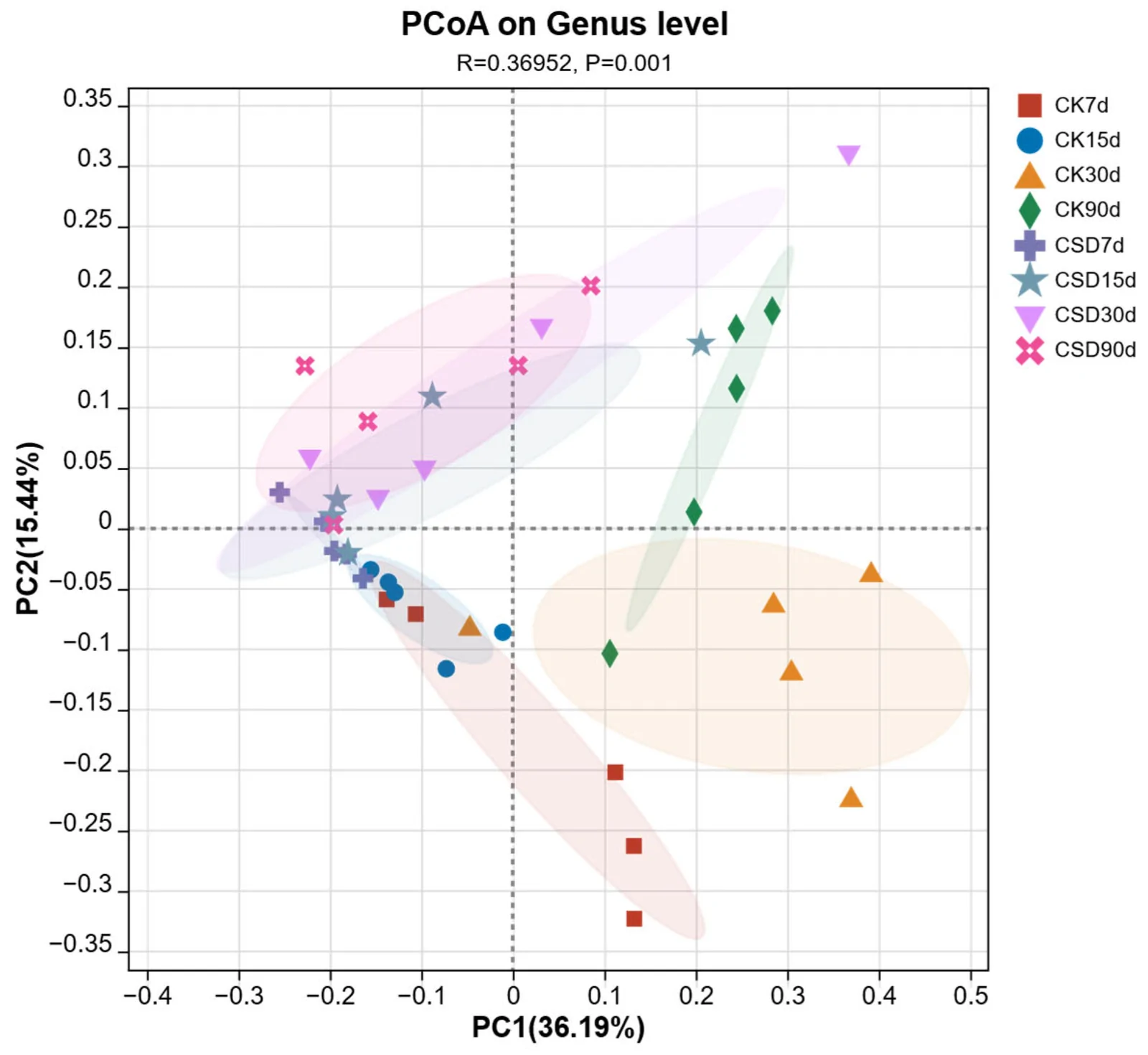
Principal coordinate analysis (PCoA) based on Bray–Curtis distances at the genus level. Note: Each point represents a single sample, colored and shaped by treatment (CK = control; CSD = 500 mg/kg TTO) and ensiling time (7, 15, 30, and 90 d). The colored ellipses represent the 95% confidence intervals for each treatment group.

PCoA separated CK and CSD at every timepoint (PERMANOVA: *R* = 0.370, *p* = 0.001); PC1 and PC2 explained 51.6% of the variation (Figure 3). CSD samples clustered more tightly than CK, suggesting less community heterogeneity under TTO selection.

#### 3.5.3. Bacterial Community Structure

**Figure 4:**
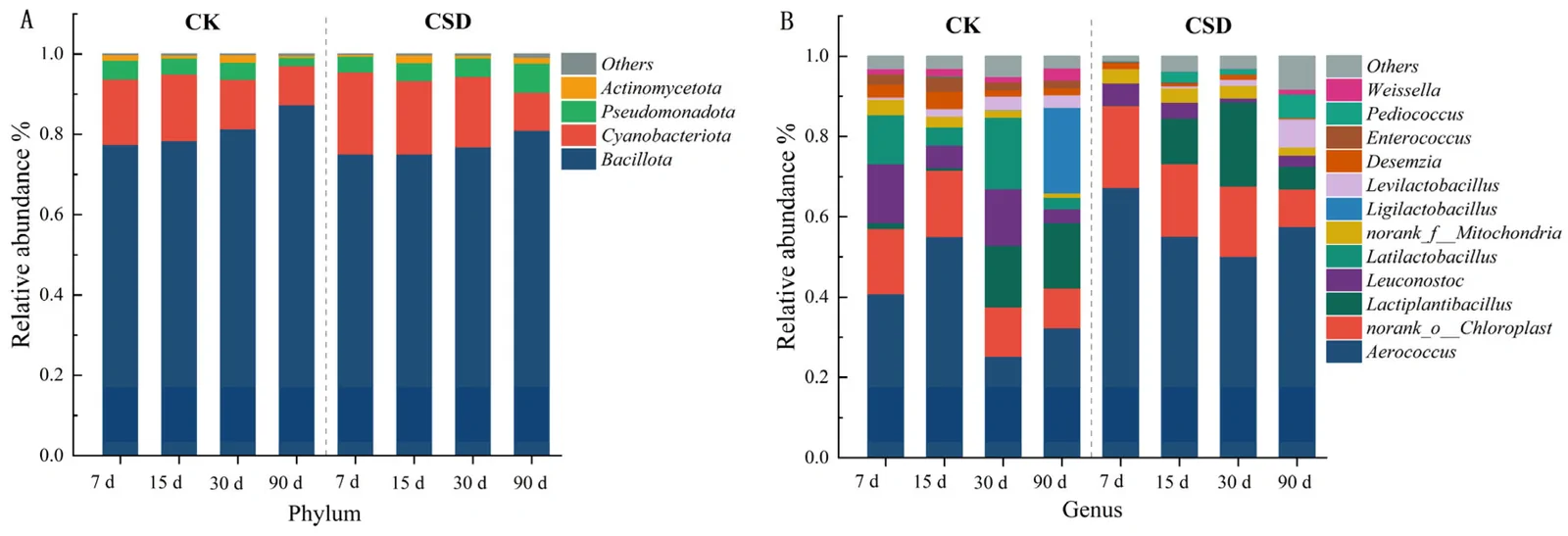
Bacterial community structure of bacterial communities in CK and CSD paper mulberry silage. (**A**) Relative abundance at the phylum level; (**B**) relative abundance at the genus level (top 12 genera with mean relative abundance > 1%). CK = control; CSD = 500 mg/kg TTO.

At the phylum level (Figure 4A), *Bacillota* dominated all samples and increased with ensiling time. At the genus level, *Aerococcus* was the most abundant genus in all samples and was higher in CSD than in CK. *Pediococcus* was consistently more abundant in CSD than in CK, reaching 5.9% on day 90 compared with only 0.04% in CK. CSD exhibited a pronounced enrichment of *Lactiplantibacillus* earlier, which constituted 20.9% of the total bacterial community at day 30, but declined to 5.6% at day 90, below the 16.2% recorded in CK. *Ligilactobacillus* was specifically enriched only in CK at day 90 (21.3%). In addition, *Latilactobacillus*, *Leuconostoc* and *Enterococcus* were more abundant in CK than in CSD.

#### 3.5.4. Correlations Between Bacterial Genera and Fermentation Indices

**Figure 5:**
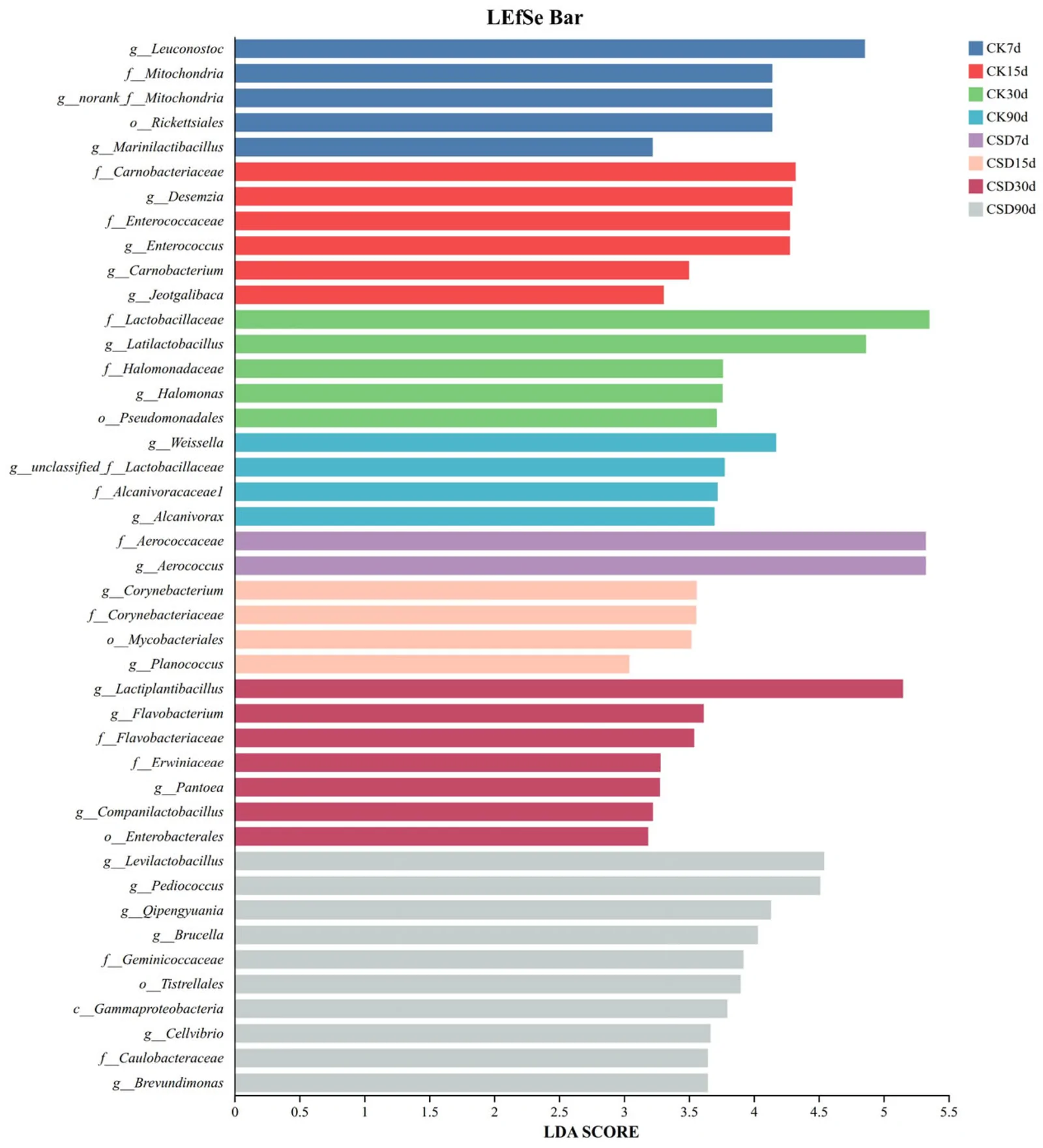
LEfSe analysis showing differentially abundant bacterial taxa (LDA score > 3.0) in paper mulberry silage. Bars represent taxa significantly enriched in CK (control) or CSD (500 mg/kg TTO) at 7, 15, 30, and 90 d of ensiling. Prefixes denote taxonomic ranks: g, genus; f, family; o, order; c, class.

LEfSe analysis (Figure 5) identified distinct biomarker taxa across the four timepoints. *Aerococcus* was the main CSD biomarker at 7 d (LDA > 5.0); *Leuconostoc* marked CK. By 30 d, *Lactiplantibacillus* dominated CSD (LDA > 5.0) while *Latilactobacillus* enriched CK. At 90 d, *Levilactobacillus* and *Pediococcus* became CSD signatures (LDA > 4.5) and *Weissella* remained the CK marker. 

**Figure 6:**
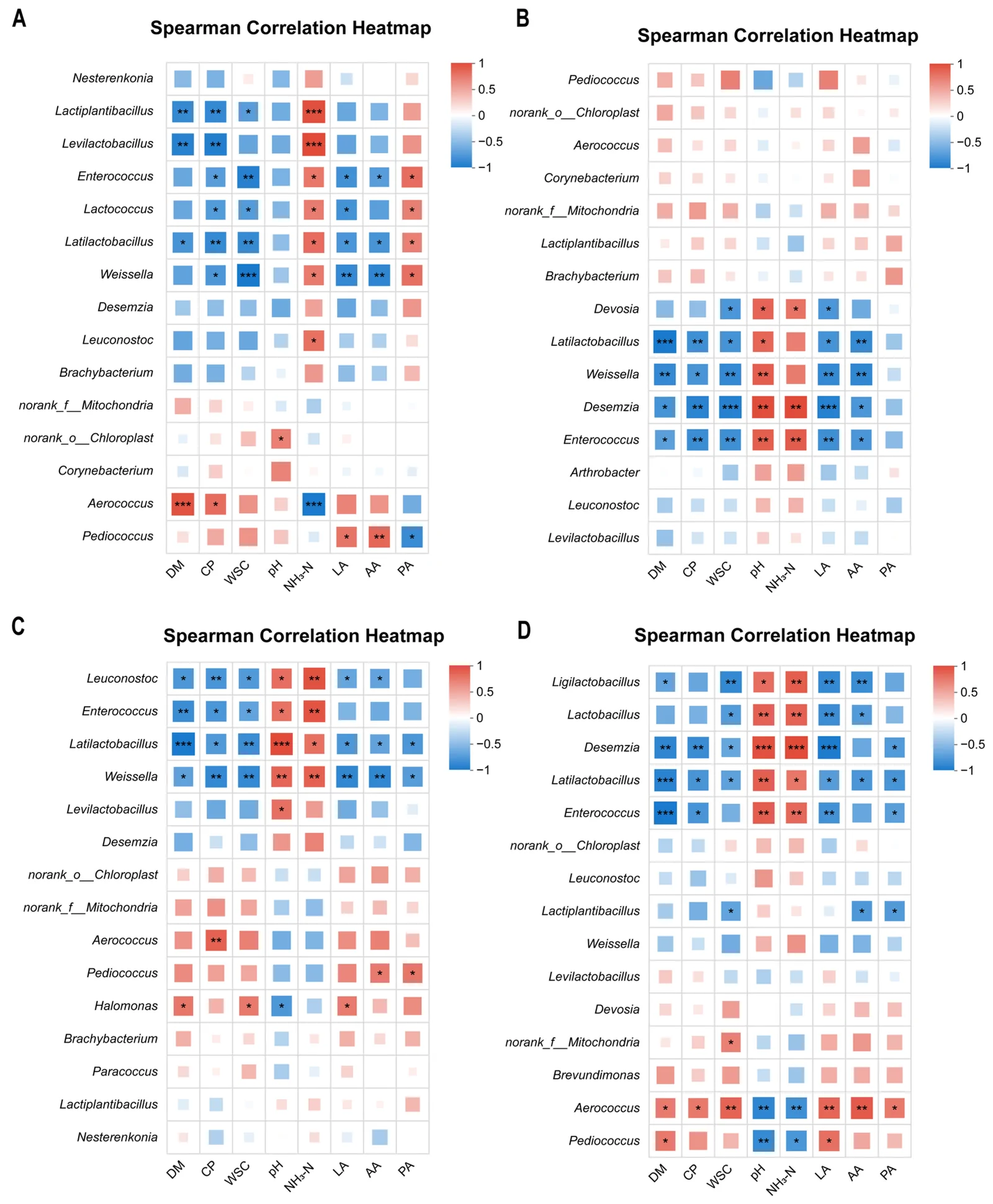
Spearman’s rank correlation heatmap between differentially abundant bacterial genera and fermentation indices during ensiling. Panels (**A**–**D**) represent the correlation relationships at 7, 15, 30, and 90 days of ensiling, respectively. Notes: Blue and red tiles indicate positive and negative correlations, respectively. The intensity of the color represents the magnitude of the correlation coefficient (r), as shown in the color scale bar. * *p* < 0.05, ** *p* < 0.01, *** *p* < 0.001. DM = dry matter; CP = crude protein; WSC = water-soluble carbohydrate; NH_3_-N = ammonia nitrogen/total nitrogen; LA = lactic acid; AA = acetic acid; PA = propionic acid.

Spearman’s correlations between bacterial genera and fermentation indices at each time point are illustrated in Figure 6. At 7 d (Figure 6A), multiple LAB genera (*Lactiplantibacillus*, *Levilactobacillus*, *Latilactobacillus*, *Enterococcus*, *Weissella*, and *Lactococcus*) were positively correlated with WSCs and LA, and negatively correlated with pH. *Aerococcus* showed an opposing pattern, correlating positively with DM, CP, and pH, but negatively with LA. At 15 d (Figure 6B), LAB genera including *Latilactobacillus*, *Weissella*, and *Enterococcus* remained positively associated with LA and AA, and negatively with pH. Devosia correlated positively with pH and negatively with LA. At 30 d (Figure 6C), the positive LA-AA correlations and negative pH correlations persisted among several LAB genera. *Enterococcus* associated positively with DM and CP but negatively with WSC, whereas *Leuconostoc* showed the opposite relationship with WSC and NH_3_-N. At 90 d (Figure 6D), the positive correlations of LAB genera with LA and AA and negative correlations with pH continued. *Aerococcus* and *Pediococcus* were positively associated with DM and CP but negatively with LA. All reported correlations were significant at *p* < 0.05.

**Figure 7:**
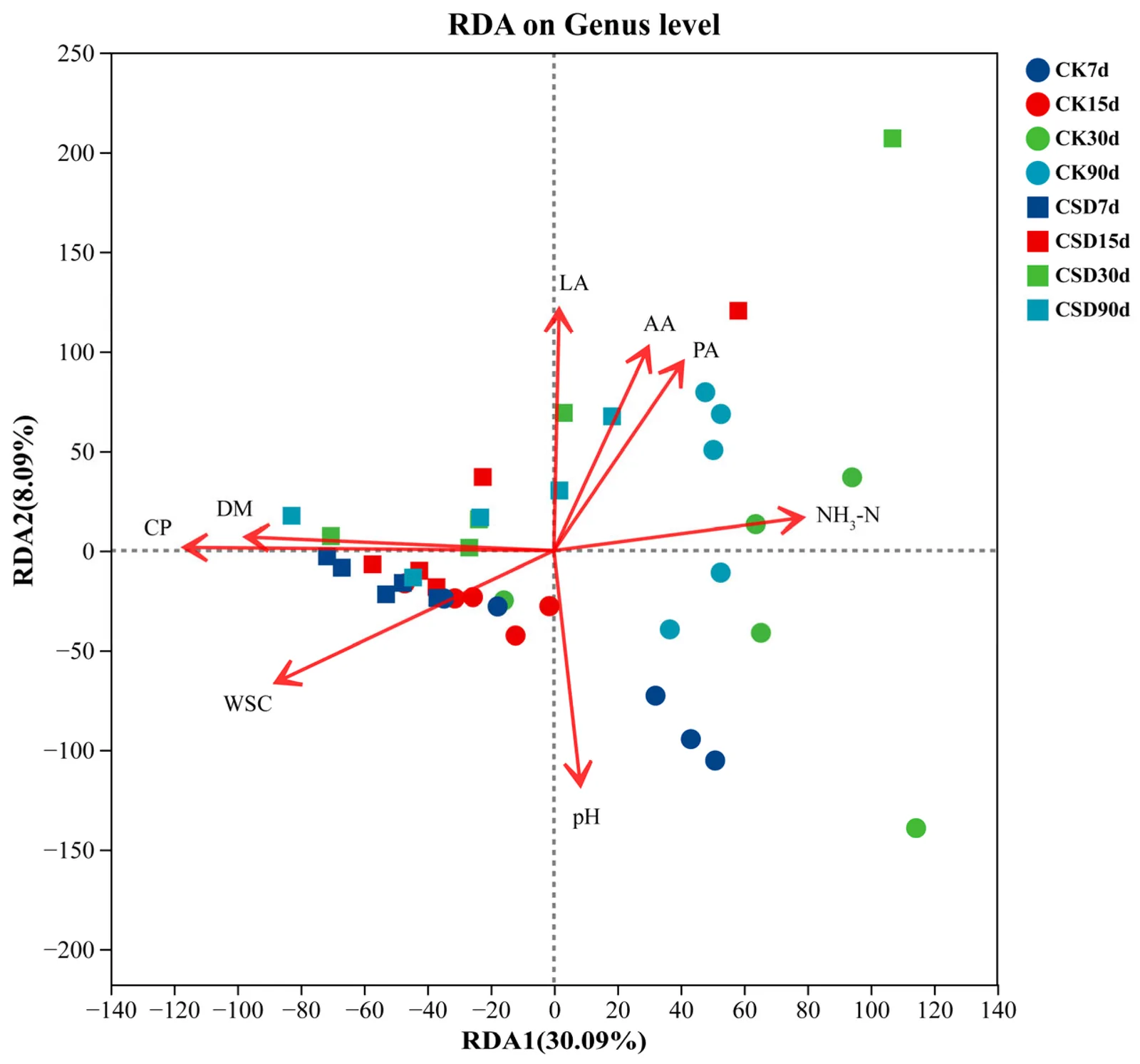
RDA of bacterial communities constrained by fermentation indices. Notes: Samples are classified by treatments (CK, CSD, and CSG) and fermentation times (7, 15, 30, and 90 days). The red vectors represent the explanatory environmental variables. The dashed lines represent the center reference lines of the RDA ordination space (x = 0 and y = 0).

RDA confirmed the covariation between bacterial community and fermentation parameters, as illustrated in Figure 7. The two groups were distinctly separated from the early stage of ensiling (7 d), and this separation persisted throughout the entire fermentation process. The first two axes cumulatively explained 38.18% of the variation. At 15 d, CSD clustered with DM, CP, and WSC, indicating rapid acidification. By 90 d, CSD shifted toward DM, AA, and PA, reflecting improved nutrient retention. In contrast, CK samples migrated toward NH_3_-N and pH vectors at 30 d and subsequently shifted toward the NH_3_-N and PA vectors at 90 d. The gap between the two groups widened over time, reaching maximum separation at 90 d.

## 4. Discussion

### 4.1. Fermentation Quality and Microbial Dynamics

The key to successful silage fermentation lies in the rapid production of lactic acid from water-soluble carbohydrates by lactic acid bacteria, which leads to a sharp decline in pH [52] and the subsequent creation of an acidic environment that inhibits the growth of spoilage microorganisms [22] and curtails proteolytic activity [53]. The pH of CSD declined rapidly during ensiling, reaching 4.20 at 90 days, the lowest among all treatments. This decline coincided with a progressive accumulation of lactic acid, which peaked by 30 days, and remained significant in CSD compared with both CK and CSG. Meanwhile, the NH_3_-N/TN ratio in CSD remained substantially below that of CK from 15 days onward. Together, these indicators demonstrate that CSD established a fermentation environment characterized by rapid acidification [54], which effectively limited protein degradation [55] and suppressed spoilage microorganisms.

The magnitude of LA accumulation in CSD can be attributed primarily to its markedly higher LAB population. Throughout ensiling, CSD consistently maintained a significantly higher LAB density than CK [56]. CSG exhibited intermediate counts, remaining higher than CK but lower than CSD, a pattern that aligns with the dose-dependent antimicrobial activity of essential oils. This substantial LAB density ensured that sufficient fermentative capacity was available to drive rapid and continuous LA production during the early stage, while sustaining high metabolic activity in the later stage as pH declined to levels that would suppressed less acid-tolerant microorganisms.

At 90 days, the LA/AA ratio in CSD was 3.04, sitting at the threshold commonly used to classify homolactic tendency [57], yet the absolute acetic acid concentration (1.85% DM) was higher than typically seen in strictly homofermentative systems [58,59]. Notably, the elevated acetic acid concentration observed in CSD occurred in the context of sufficient lactic acid accumulation (5.63% DM) and effective pH reduction (4.20), a pattern fundamentally different from the passive acetic acid production typically associated with poor fermentation in high-moisture silage [60,61]. This profile reflects the dual differences between the two systems in both the absolute abundance and relative community structure of LAB. Although the relative proportion of the homofermentative genus *Lactiplantibacillus* was even higher in CK than in CSD, the total LAB counts in CK were significantly lower. In contrast, CSD, leveraging its overwhelmingly larger LAB baseline, not only maintained high abundances of homofermentative genera (predominantly *Lactiplantibacillus* and *Pediococcus*) [62] but also of heterofermentative genera (*Levilactobacillus*) [63], whereas the CK community was predominantly occupied by heterofermentative or facultatively heterofermentative genera (*Ligilactobacillus* and *Latilactobacillus*) [64,65,66,67,68]. This compositional difference has direct metabolic consequences: homofermentative populations convert glucose almost quantitatively to lactic acid without CO_2_ production [62,63], enabling rapid acidification and efficient substrate conservation, whereas heterofermentative populations generate acetic acid and CO_2_ alongside lactic acid [66], slowing pH decline and increasing dry matter loss [67].

In CSD, homofermentative populations dominated the early phase of ensiling, driving the rapid pH decline documented above. The subsequent enrichment of heterofermentative populations in the later stage contributed additional AA, but because this occurred on the high baseline of an already abundant LAB population, it did not compromise the primary LA pathway. The result was a fermentation profile that combined the rapid acidification typical of homofermentative systems with the antifungal benefits of moderate acetic acid, all while maintaining LA/AA near 3.04. By contrast, the prolonged heterofermentative predominance in CK produced slower acidification, greater substrate loss via CO_2_, and higher proteolysis, as reflected by the elevated NH_3_-N/TN ratio, ultimately yielding a poorer preserved silage.

The microbial community responded to TTO in a trade-off manner, where increasing the concentration from 500 to 1000 mg/kg did not further enhance, but rather attenuated, the beneficial modulation of LAB populations. CSD achieved superior fermentation outcomes compared with CSG, as reflected by lower terminal pH and higher LAB counts. This suggests that the higher concentration of essential oil in CSG may have exerted inhibitory effects on LAB, offsetting its antimicrobial benefits against spoilage organisms, a pattern consistent with the dose-dependent antimicrobial activity of plant essential oils reported previously [18]. In CSD, mold counts remained consistently and significantly lower than those in CK throughout the ensiling period. Meanwhile, the concurrent rise in LAB indicates that CSD created a selective environment where acid-tolerant LAB outcompeted pH-sensitive spoilage organisms [53]. The absence of this effect in CSG, which showed intermediate pH and lower LAB counts than CSD, supports a nonlinear dose–response relationship for TTO in paper mulberry silage, as has been observed for other essential oils in silage systems [23,68].

### 4.2. In Vitro Gas Production Characteristics

The cumulative gas production profiles were consistent with the Gompertz model-derived kinetic parameters. The theoretical maximum gas production, gas production rate, and gas production lag time can indicate the availability and fermentability of substrates to rumen microorganisms [69,70]. In the present study, TTO treatments significantly accelerated gas production kinetics, as reflected by the higher *C*_3_ rate parameters compared with CK. The theoretical maximum gas production was significantly enhanced in CSD, exceeding both CK and CSG, suggesting that CSD enlarged the total fermentable substrate pool. However, the markedly faster gas production rates and the shortest *λ* observed in CSD indicate that the substrate matrix in CSD was more rapidly fermentable [71], a feature most likely attributable to better preservation of readily fermentable carbohydrates during ensiling. This interpretation aligns with the higher WSC content preserved in the TTO treatments at 90 days.

Although CSG showed a comparable *C*_3_ to CSD, its markedly prolonged *λ* and *Tmax* indicate that the higher essential oil concentration may have initially inhibited rumen microbial activity, thereby delaying the onset of fermentation. Consequently, CSD, at the lower dosage, consistently exhibited marginally better values across *C*_1_, *C*_3_, and *λ*. The combination of a significantly larger total gas production with accelerated fermentation kinetics [69,70,71] implies that TTO addition not only increased the quantity of fermentable substrate but also enhanced its availability and utilization rate by rumen microorganisms. This pattern is consistent with the view that improved silage preservation, characterized by rapid acidification and reduced microbial spoilage, preserves the structural and chemical integrity of fermentable substrates, thereby facilitating faster rumen fermentation without altering the absolute substrate pool [72].

Notably, the highest *C*_1_ in CSD occurred despite its homolactic-dominant fermentation (LA/AA = 3.04), indicating that the efficient preservation of substrates during ensiling, rather than the fermentation type itself, determines the subsequent rumen fermentability [73].

### 4.3. Bacterial Community Structure and Functional Implications

The application of TTO fundamentally restructured the bacterial community of paper mulberry silage. At 7, 15, and 30 days, Shannon diversity was consistently and significantly higher in CK than in CSD, yet CK exhibited inferior fermentation quality. This suggests that TTO inhibited the relative abundance of sensitive groups, and thus beneficial functional groups are effectively enriched under selective pressure, thereby dominating the fermentation process and improving silage quality [74,75]. Rather, the selective pressure exerted by the essential oil suppressed less competitive taxa, resulting in a streamlined community with lower evenness but higher functional specificity [37]. Shannon and Chao1 indices exhibited distinct response patterns during this period. Given that Shannon index primarily reflects species evenness, whereas Chao1 index mainly reflects species richness, their differential responses to selective pressure further corroborate the complementary nature of these two indices in ecological interpretation. During the first 30 days, Chao1 richness remained comparable between treatments; this may be because the suppressive effect of TTO on sensitive taxa was offset by the rapid proliferation of acid-tolerant lactic acid bacteria that occupied vacant niches. By 90 days, Shannon indices had converged between CK and CSD, while CSD showed greater Chao1 richness at the same time point. This may be because additional taxa colonized once the acidic environment had stabilized [76].

Principal coordinate analysis (PCoA) provided further evidence of community restructuring, showing a clear and consistent separation between CK and CSD bacterial communities across all four time points. This sustained divergence confirms that TTO did not merely suppress microbial growth, but actively reshaped community composition from the outset of ensiling [77,78,79].

Although *Bacillota* dominated all samples and is typically dominant in other silages as well [80], this relative at the phylum level masked a functionally significant reorganization occurring at finer taxonomic scales. The genus-level community composition revealed that this structural divergence was driven primarily by differential enrichment of specific LAB genera between the two treatments, as detailed below.

CSD showed the temporal succession, from homofermentative *Lactiplantibacillus* at 30 d to a mixed population of heterofermentative *Levilactobacillus* and homofermentative *Pediococcus* at 90 d. This successional pattern provides statistical confirmation of the functional group dynamics: rapid initial acidification by homofermentative LAB, followed by sustained low pH with moderate acetic acid production from heterofermentative activity.

Spearman correlation analysis across the four timepoints validated the functional relevance of the community shifts identified by LEfSe. The consistent positive correlations of *Lactiplantibacillus* with LA, and its negative correlations with pH, confirm that this genera was a metabolically active contributor to acidification rather than a mere bystander [79,80] enriched by the acidic environment. This finding is consistent with the known metabolic traits of these genera [65,66,68] and provides statistical confirmation that the LEfSe-identified biomarkers were functionally relevant to the key chemical changes driving fermentation quality.

*Aerococcus* presented a distinct ecological pattern. Despite being the most abundant genus in early-stage CSD (67%), it correlated positively with DM and CP and was also positively correlated with LA and AA. This suggests that *Aerococcus* may have capitalized on the nutrient-rich substrates available at the onset of ensiling and co-existed with the acidic environment, rather than acting as a primary acid production. This differs from a previous report in which *Aerococcus* was negatively correlated with AA in paper mulberry silage [81], suggesting that the presence of TTO may have shifted its ecological association. Its high abundance, despite lacking significant negative correlations with acidification, raises the possibility that it functions as an early colonizer tolerant of the selective environment created by TTO; however, its metabolic role, whether as a commensal, nutrient competitor, or passive occupant, remains unresolved and warrants further investigation using metagenomic or culture-based approaches.

Redundancy analysis (RDA) provided a community-level perspective that complemented these genus-specific correlations. The separation of CK and CSD samples along the RDA1 axis, with CK shifting toward pH and NH_3_-N and CSD toward AA, PA, WSCs, DM, and CP, indicates that the essential oil redirected the community’s functional output toward acidification and nutrient preservation and the accumulation of fermentation products. This community-level shift is consistent with the mechanism proposed by Hao et al. [31], who suggested that essential oils exert selective pressure on microbial populations, thereby improving nutrient conservation and fermentation quality. Our multi-timepoint RDA further extends this framework by revealing two novel features. First, the progressive widening of the gap between CK and CSD over time demonstrates that this selective pressure was continuously reinforced rather than a one-time event, indicating a sustained steering of the CSD community toward high-quality fermentation. Second, the association of CSD with multiple beneficial parameters (WSC, DM, CP, AA and PA) in a single ordination space further suggests that these improvements were covariant. They arose from the same community-level functional shift, rather than from independent, unrelated microbial activities.

## 5. Conclusions

TTO at 500 mg/kg (CSD) improved fermentation quality, nutrient preservation, and rumen fermentability of paper mulberry silage. This was achieved through rapid acidification, a favorable LA/AA ratio of approximately 3.04, and the temporal selective enrichment of functional LAB genera, transitioning from *Lactiplantibacillus* to *Pediococcus*, and *Levilactobacillus*. The higher dose (1000 mg/kg) was less effective, suggesting an upper inhibitory threshold. The role of *Aerococcus* in the early fermentation stage and the in vitro nature of rumen assays warrant further investigation. Overall, CSD at 500 mg/kg is a promising natural additive for paper mulberry silage.

## Figures and Tables

**Table 1:** Nutritional composition of paper mulberry raw material (dry matter basis).

Item	Content
DM, % FW	32.91 ± 0.19
CP, % DM	21.48 ± 0.14
WSC, % DM	8.80 ± 0.19
NDF, % DM	45.69 ± 1.71
ADF, % DM	29.11 ± 0.88

Footnote: FW = fresh weight; DM = dry matter; CP = crude protein; WSC = water-soluble carbohydrate; NDF = neutral detergent fiber; ADF = acid detergent fiber. Values are presented as mean ± SD (*n* = 3).

**Table 2:** Effects of treatment and ensiling time on nutritional composition of paper mulberry silage.

Item	Time	CK	CSD	CSG	SEM	*p* Value		
						Treatment	Time	Treatment * Time
DM, %FW	7 d	27.50 ^Ab^	28.83 ^Aa^	27.71 ^Ab^	0.292	<0.001	<0.001	<0.001
	15 d	24.61 ^Bb^	27.95 ^Ba^	24.23 ^Bb^				
	30 d	22.85 ^Cc^	27.81 ^Ba^	24.11 ^Bb^				
	90 d	21.79 ^Dc^	25.55 ^Ca^	23.36 ^Bb^				
CP, %DM	7 d	19.06 ^ABb^	21.35 ^Aa^	20.42 ^Aa^	0.362	<0.001	<0.001	0.399
	15 d	19.70 ^Aa^	20.37 ^ABa^	20.03 ^Aa^				
	30 d	18.31 ^BCb^	19.92 ^Ba^	19.55 ^ABa^				
	90 d	17.32 ^Cb^	19.30 ^Ba^	18.82 ^Ba^				
WSC, %DM	7 d	8.55 ^Ab^	9.30 ^Aa^	9.29 ^Aa^	0.165	<0.001	<0.001	0.201
	15 d	6.17 ^Bb^	6.97 ^Ba^	6.75 ^Ba^				
	30 d	3.82 ^Cb^	4.85 ^Ca^	5.00 ^Ca^				
	90 d	3.65 ^Cb^	5.13 ^Ca^	4.90 ^Ca^				
NDF, %DM	7 d	51.05 ^Aa^	51.01 ^Aa^	50.58 ^Aa^	0.365	0.004	<0.001	0.008
	15 d	49.20 ^Ba^	49.57 ^Ba^	48.98 ^Ba^				
	30 d	45.90 ^Cb^	48.24 ^Ca^	48.39 ^Ba^				
	90 d	45.12 ^Cb^	46.36 ^Da^	45.56 ^Cab^				
ADF, %DM	7 d	28.56 ^Aa^	28.84 ^Aa^	28.73 ^Aa^	0.135	<0.001	<0.001	0.633
	15 d	28.00 ^Bb^	28.44 ^Ba^	28.51 ^Aa^				
	30 d	27.32 ^Cb^	27.87 ^Ca^	27.54 ^Bab^				
	90 d	26.58 ^Db^	27.20 ^Da^	26.91 ^Cab^				

Footnote: CK = control; CSD = 500 mg/kg TTO; CSG = 1000 mg/kg TTO. * Treatment × Time interaction (two-way ANOVA). ^A–D^ Means within a column (same treatment across different times) with different uppercase superscripts differ (*p* < 0.05). ^a–c^ Means within a row (same time across different treatments) with different lowercase superscripts differ (*p* < 0.05). Data are presented as means; SEM represents the pooled standard error of the mean for the interaction between treatment and time.

**Table 3:** Effects of treatment and ensiling time on fermentation characteristics and organic acid content of paper mulberry silage.

Item	Time	CK	CSD	CSG	SEM	*p* Value		
						Treatment	Time	Treatment * Time
pH	7 d	6.89 ^Aa^	6.91 ^Aa^	6.41 ^Aa^	0.189	<0.001	<0.001	<0.001
	15 d	6.55 ^Aa^	5.23 ^Bb^	6.18 ^Aa^				
	30 d	6.33 ^Aa^	4.78 ^Bb^	6.11 ^Aa^				
	90 d	5.64 ^Ba^	4.20 ^Cb^	5.19 ^Ba^				
NH_3_-N/TN, %	7 d	3.78 ^Ca^	2.13 ^Ca^	2.55 ^Ca^	0.687	<0.001	<0.001	0.008
	15 d	9.34 ^Ba^	5.13 ^Bb^	4.95 ^Bb^				
	30 d	10.40 ^Ba^	4.55 ^Bb^	5.79 ^ABb^				
	90 d	14.42 ^Aa^	9.20 ^Ab^	7.40 ^Ab^				
LA, %DM	7 d	1.30 ^Cb^	2.30 ^Ca^	1.43 ^Cb^	0.225	<0.001	<0.001	0.0175
	15 d	2.23 ^Bb^	3.70 ^Ba^	2.71 ^Bb^				
	30 d	3.37 ^Ac^	6.09 ^Aa^	4.86 ^Ab^				
	90 d	3.72 ^Ac^	5.63 ^Aa^	4.60 ^Ab^				
AA, %DM	7 d	0.47 ^Dab^	0.58 ^Ca^	0.44 ^Db^	0.039	<0.001	<0.001	<0.001
	15 d	0.78 ^Cb^	0.96 ^Ba^	0.96 ^Ca^				
	30 d	1.26 ^Bc^	1.79 ^Aa^	1.42 ^Bb^				
	90 d	1.46 ^Ab^	1.85 ^Aa^	1.78 ^Aa^				
PA, %DM	7 d	0.10 ^Ba^	0.09 ^Ba^	0.07 ^Ba^	0.120	<0.001	<0.001	0.002
	15 d	0.25 ^Ba^	0.30 ^Ba^	0.30 ^Ba^				
	30 d	0.93 ^Ab^	1.38 ^Aa^	1.12 ^Aab^				
	90 d	1.08 ^Ab^	1.64 ^Aa^	1.45 ^Aa^				

Footnote: * Treatment × Time interaction (two-way ANOVA). NH_3_-N/TN = ammonia nitrogen/total nitrogen; LA = lactic acid; AA = acetic acid; PA = propionic acid. ^A–D^ Means within a column (same treatment across different times) with different uppercase superscripts differ (*p* < 0.05). ^a–c^ Means within a row (same time across different treatments) with different lowercase superscripts differ (*p* < 0.05). Data are presented as means; SEM represents the pooled standard error of the mean for the interaction between treatment and time.

**Table 4:** Effects of treatment and ensiling time on microbial populations of paper mulberry silage.

Item	Time	CK	CSD	CSG	SEM	*p* Value		
						Treatment	Time	Treatment * Time
LAB Log_10_ CFU/g FM	7 d	7.19 ^Aa^	7.33 ^Ca^	7.26 ^Ca^	0.058	<0.001	<0.001	<0.001
	15 d	6.93 ^Bb^	7.46 ^Ca^	7.33 ^Ca^				
	30 d	6.34 ^Cb^	8.22 ^Ba^	8.08 ^Ba^				
	90 d	6.15 ^Dc^	8.75 ^Aa^	8.34 ^Ab^				
Mold Log_10_ CFU/g FM	7 d	4.29 ^Aa^	4.22 ^Ab^	4.24 ^Aab^	0.021	<0.001	<0.001	<0.001
	15 d	4.13 ^Ba^	4.02 ^Bb^	4.03 ^Bb^				
	30 d	4.08 ^Ba^	3.49 ^Cc^	3.59 ^Cb^				
	90 d	3.86 ^Ca^	3.25 ^Dc^	3.47 ^Db^				
Yeast Log_10_ CFU/g FM	7 d	4.39 ^Da^	3.77 ^Dc^	3.99 ^Db^	0.026	<0.001	<0.001	<0.001
	15 d	5.12 ^Ca^	4.53 ^Cc^	4.63 ^Cb^				
	30 d	5.27 ^Ba^	5.16 ^Bb^	5.17 ^Bb^				
	90 d	5.53 ^Aa^	5.45 ^Aa^	5.47 ^Aa^				
AB Log_10_ CFU/g FM	7 d	7.14 ^Ab^	7.63 ^Aa^	7.56 ^Aa^	0.032	<0.001	<0.001	<0.001
	15 d	7.00 ^Ba^	6.76 ^Bb^	6.96 ^Ba^				
	30 d	6.85 ^Ca^	6.43 ^Cc^	6.64 ^Cb^				
	90 d	6.76 ^Ca^	6.37 ^Cb^	6.36 ^Db^				

Footnote: * Treatment × Time interaction (two-way ANOVA). LAB = lactic acid bacteria; Mold = mold count; AB = aerobic bacteria; CFU/g FM = colony-forming units per gram of fresh matter. ^A–D^ Means within a column (same treatment across different times) with different uppercase superscripts differ (*p* < 0.05). ^a–c^ Means within a row (same time across different treatments) with different lowercase superscripts differ (*p* < 0.05). Data are presented as means; SEM represents the pooled standard error of the mean for the interaction between treatment and time.

**Table 5:** Gompertz model parameters of in vitro gas production kinetics.

Treatment	CK	CSD	CSG	*p* Value
*C*_1_, mL·200 mg^−1^ DM	18.27 ± 0.27 ^c^	25.13 ± 1.87 ^a^	22.16 ± 0.42 ^b^	<0.001
*C*_2_	3.31 ± 0.38 ^b^	6.23 ± 1.92 ^b^	15.24 ± 6.25 ^a^	0.02
*C*_3_, h^−1^	0.070 ± 0.010 ^b^	0.182 ± 0.028 ^a^	0.229 ± 0.085 ^a^	0.024
*λ*, h	33.26 ± 4.84 ^b^	28.20 ± 5.81 ^b^	61.13 ± 11.95 ^a^	0.005
*Tmax*, h	47.78 ± 5.65 ^b^	33.78 ± 5.04 ^b^	65.87 ± 11.28 ^a^	0.007

Footnote: *C*_1_, the theoretical maximum gas production (mL·200 mg^−1^ DM); *C*_2_, the gas production rate parameter; *C*_3_, the gas production rate constant (h^−1^); *λ*, the lag time (h); *Tmax*, the time to reach the maximum gas production rate (h). Values are expressed as mean ± standard deviation (*n* = 3). ^a–c^ means within the same row with different superscript lowercase letters are significantly different (Duncan’s multiple range test, *p* < 0.05). *p*-values were derived from one-way ANOVA.

## Data Availability

The data presented in this study are available upon request from the first author. Data supporting the bacterial community in the Sequence Read Archive (SRA) can be found at the National Center for Biotechnology Information (NCBI). SRA number: PRJNA1508728.

## Supplementary Materials

The following supporting information can be downloaded at https://www.mdpi.com/article/10.3390/microorganisms14091957/s1, Table S1: Chemical composition, fermentation quality, and microbial counts of silage across different treatment groups after 90 days of ensiling.

## Abbreviations

The following abbreviations are used in this manuscript:

AA	Acetic acid
ADF	Acid detergent fiber
BA	Butyric acid
CK	Control group
CP	Crude protein
CSD	500 mg/kg tea tree essential oil
CSG	1000 mg/kg tea tree essential oil
DM	Dry matter
EE	Ether extract
LA	Lactic acid
LAB	Lactic acid bacteria
NDF	Neutral detergent fiber
NH_3_-N	Ammoniacal nitrogen
PA	Propionic acid
WSC	Water-soluble carbohydrate
