# Supplementary material for: Tea Tree Essential Oil Enriches Lactic Acid Bacteria and Improves Paper Mulberry Silage Quality
Source: Microorganisms. 2026 Sep 4;14(9):1957. doi: 10.3390/microorganisms14091957 (PMC13609118; doi:10.3390/microorganisms14091957)
Supplement: Supplementary file 1 [file microorganisms-14-01957-s001.zip › microorganisms-4522999-supplementary.pdf]

Table S1. Chemical composition, fermentation quality, and microbial counts of silage across different treatment groups after 90 days of ensiling.

Treatment	DM, %FW	CP, %DM	WSC, %DM	NDF, %DM	ADF, %DM	pH	NH_3-N/TN, %	LA, %DM	AA, %DM	PA, %DM	LAB log_{10}CFU/g FM	Mold log_{10}CFU/g FM	Yeast log_{10}CFU/g FM	AB log_{10}CFU/g FM
CK	21.79 ± 0.74^{e}	17.32 ± 0.13	3.65 ± 0.30^{b}	45.12 ± 0.67^{bcd}	26.58 ± 0.13	5.64 ± 0.63^{a}	14.42 ± 1.29^{a}	3.72 ± 0.35	1.46 ± 0.04^{g}	1.08 ± 0.06	6.15 ± 0.06^{e}	3.86 ± 0.09^{a}	5.53 ± 0.03	6.76 ± 0.02^{a}
SZD	23.69 ± 0.60^{cd}	17.98 ± 0.42	4.98 ± 0.36^{a}	45.98 ± 0.45^{abc}	26.83 ± 0.02	5.18 ± 0.52^{ab}	11.08 ± 2.41^{b}	4.9 ± 0.58	1.82 ± 0.1^{ab}	1.26 ± 0.19	8.41 ± 0.06^{b}	3.48 ± 0.03^{b}	5.53 ± 0.03	6.37 ± 0.01^{b}
SZG	24.41 ± 2.22^{bc}	18.52 ± 1.19	4.90 ± 0.35^{a}	46.14 ± 0.56^{abc}	26.86 ± 0.12	4.98 ± 0.35^{ab}	7.19 ± 0.72^{c}	4.28 ± 1.1	1.69 ± 0.07^{bcde}	1.38 ± 0.42	8.49 ± 0.04^{ab}	3.48 ± 0.01^{b}	5.54 ± 0.05	6.43 ± 0.03^{b}
HJD	23.42 ± 0.38^{cd}	18.54 ± 0.08	4.91 ± 0.24^{a}	45.16 ± 0.67^{bcd}	26.91 ± 0.15	4.91 ± 0.17^{ab}	8.19 ± 0.6^{bc}	4.39 ± 0.27	1.67 ± 0.04^{cde}	1.16 ± 0.07	8.46 ± 0.09^{b}	3.46 ± 0.01^{b}	5.48 ± 0.06	6.4 ± 0.04^{b}
HJG	21.79 ± 0.74^{e}	17.84 ± 0.05	4.81 ± 0.24^{a}	46.38 ± 0.21^{ab}	27.12 ± 0.45	5.09 ± 0.38^{ab}	9.53 ± 2.17^{bc}	4.36 ± 0.84	1.53 ± 0.03^{fg}	1.51 ± 0.28	8.43 ± 0.11^{b}	3.46 ± 0.02^{b}	5.48 ± 0.11	6.38 ± 0.04^{b}
BLXD	24.47 ± 0.10^{bc}	19.21 ± 0.31	4.86 ± 0.41^{a}	46.68 ± 0.27^{a}	27.06 ± 0.39	4.42 ± 0.03^{b}	7.4 ± 0.75^{c}	5.36 ± 1.43	1.72 ± 0.12^{bcd}	1.38 ± 0.16	8.42 ± 0.04^{b}	3.49 ± 0.03^{b}	5.46 ± 0.09	6.37 ± 0.05^{b}
BLXG	25.31 ± 0.16^{ab}	18.88 ± 0.49	4.89 ± 0.45^{a}	45.35 ± 0.57^{abcd}	27.00 ± 0.45	4.59 ± 0.12^{b}	8.82 ± 2.07^{bc}	5.02 ± 1.61	1.52 ± 0.02^{fg}	1.53 ± 0.34	8.4 ± 0.04^{b}	3.44 ± 0.04^{b}	5.46 ± 0.05	6.36 ± 0.04^{b}
CSD	25.55 ± 0.5^{ab}	19.30 ± 0.13	5.13 ± 0.39^{a}	46.36 ± 0.08^{ab}	27.20 ± 0.20	4.2 ± 0.08^{c}	9.2 ± 1.38^{bc}	5.63 ± 0.48	1.85 ± 0.13^{a}	1.64 ± 0.29	8.75 ± 0.16^{a}	3.25 ± 0.02^{c}	5.45 ± 0.04	6.13 ± 0.06^{d}
CSG	23.36 ± 0.34^{cd}	18.82 ± 0.89	4.90 ± 0.32^{a}	45.56 ± 0.62^{abcd}	26.91 ± 0.27	5.19 ± 0.43^{ab}	7.4 ± 1.86^{c}	4.6 ± 0.4	1.78 ± 0.1^{abc}	1.45 ± 0.31	8.34 ± 0.11^{bcd}	3.47 ± 0.05^{b}	5.47 ± 0.05	6.31 ± 0.04^{b}
NZD	24.98 ± 0.89^{ab}	17.70 ± 0.47	4.95 ± 0.23^{a}	46.38 ± 1.04^{ab}	26.90 ± 0.14	5.04 ± 0.1^{ab}	9.98 ± 2.69^{bc}	4.62 ± 0.92	1.65 ± 0.08^{def}	1.23 ± 0.18	8.38 ± 0.1^{bc}	3.46 ± 0.04^{b}	5.47 ± 0.05	6.34 ± 0.03^{b}
NZG	23.64 ± 0.93^{cd}	18.49 ± 0.88	4.87 ± 0.29^{a}	45.22 ± 0.84^{bcd}	26.80 ± 0.04	4.45 ± 0.22^{b}	7.87 ± 0.39^{bc}	4.34 ± 0.15	1.61 ± 0.07^{def}	1.41 ± 0.24	8.47 ± 0.14^{b}	3.4 ± 0.14^{bc}	5.48 ± 0.1	6.41 ± 0.08^{b}
MGD	22.93 ± 0.53^{d}	18.23 ± 0.17	4.86 ± 0.48^{a}	45.90 ± 0.63^{abc}	26.88 ± 0.18	4.54 ± 0.1^{b}	10.45 ± 0.86^{bc}	4.05 ± 0.54	1.57 ± 0.05^{efg}	1.43 ± 0.08	8.43 ± 0.08^{b}	3.49 ± 0.04^{b}	5.47 ± 0.09	6.38 ± 0.02^{b}
MGG	25.15 ± 0.45^{ab}	19.01 ± 0.96	4.49 ± 0.05^{a}	44.40 ± 1.35^{d}	27.26 ± 0.26	4.29 ± 0.07^{b}	7.27 ± 1.11^{c}	5.12 ± 0.44	1.58 ± 0.02^{efg}	1.28 ± 0.17	8.13 ± 0.05^{cd}	3.49 ± 0.03^{b}	5.45 ± 0.04	6.15 ± 0.02^{cd}
ZRD	25.72 ± 0.19^{a}	17.99 ± 1.82	4.88 ± 0.44^{a}	44.86 ± 1.39^{cd}	26.90 ± 0.47	4.31 ± 0.05^{b}	7.67 ± 1.07^{c}	5.44 ± 0.49	1.56 ± 0.03^{efg}	1.33 ± 0.27	8.5 ± 0.06^{ab}	3.43 ± 0.03^{b}	5.52 ± 0.09	6.43 ± 0.04^{b}
ZRG	21.79 ± 0.62^{e}	17.83 ± 0.10	4.78 ± 0.30^{a}	45.45 ± 0.37^{abcd}	27.07 ± 0.17	4.97 ± 0.12^{ab}	10.12 ± 3.11^{bc}	4.24 ± 0.17	1.56 ± 0.03^{efg}	1.55 ± 0.43	8.1 ± 0.04^{d}	3.43 ± 0.05^{b}	5.46 ± 0.09	6.62 ± 0.17a
P-value	< 0.001	0.058	0.005	0.025	0.316	< 0.001	< 0.001	0.166	< 0.001	0.421	< 0.001	< 0.001	0.853	< 0.001

Footnote: CK, control (no additive); SZD, 500 mg/kg pine needle essential oil; SZG, 1000 mg/kg pine needle essential oil; HJD, 500 mg/kg Sichuan pepper essential oil; HJG, 1000 mg/kg Sichuan pepper essential oil; BLXD, 500 mg/kg thyme essential oil; BLXG, 1000 mg/kg thyme essential oil; CSD, 500 mg/kg tea tree essential oil; CSG, 1000 mg/kg tea tree essential oil; NZD, 500 mg/kg oregano essential oil; NZG, 1000 mg/kg oregano essential oil; MGD, 500 mg/kg rose essential oil; MGG, 1000 mg/kg rose essential oil; ZRD, 500 mg/kg cumin essential oil; ZRG, 1000 mg/kg cumin essential oil. DM, dry matter; FW, fresh weight; CP, crude protein; WSC, water-soluble carbohydrates; NDF, neutral detergent fiber; ADF, acid detergent fiber; NH_3-N/TN, ammonia nitrogen to total nitrogen ratio; LA, lactic acid; AA, acetic acid; PA, propionic acid; LAB, lactic acid bacteria; FM, fresh matter; AB, aerobic bacteria. All values are presented as mean ± standard deviation (n = 3).
